# Prevalence of amyotrophic lateral sclerosis in Latium region, Italy

**DOI:** 10.1002/brb3.2378

**Published:** 2021-10-30

**Authors:** Maria Puopolo, Ilaria Bacigalupo, Paola Piscopo, Eleonora Lacorte, Alessandra Di Pucchio, Marialaura Santarelli, Maurizio Inghilleri, Antonio Petrucci, Mario Sabatelli, the Latium Registry Group, Nicola Vanacore

**Affiliations:** ^1^ Department of Neuroscience National Institute of Health Rome Italy; ^2^ National Center for Disease Prevention and Health Promotion National Institute of Health Rome Italy; ^3^ Presidio Ospedaliero San Filippo Neri ‐ ASL RM1 Rome Italy; ^4^ Umberto I Policlinico Universitario Rome Italy; ^5^ S. Camillo Forlanini Rome Italy; ^6^ Fondazione Policlinico Universitario A. Gemelli IRCCS Rome Italy

**Keywords:** amyotrophic lateral sclerosis, epidemiology studies, neurodegenerative, prevalence, public health

## Abstract

**Objective:**

Prevalence estimate of amyotrophic lateral sclerosis (ALS) ranged between 1.1/100,000 and 11.2/100,000 inhabitants with different design of the study (prospective or retrospective) and sample size. The aim of this study is to conduct for the first time an estimate of the ALS prevalence in the Latium region.

**Materials and methods:**

The study was performed in Latium, a region located in the center of Italy, with a population, as of January 1, 2016, of 5888.472 inhabitants. In this region, a network of 15 clinical centers (of which 4 referral ALS centers are located in Rome) and 10 local health authorities involved in the diagnosis and treatment of ALS patients has been identified. Each patient was classified according to the El Escorial revised criteria.

**Results:**

The prevalence study in 2016 identified 353 ALS cases (200 males). By considering population aged >=20 years, the total crude prevalence rate resulted 7.33 (CI95% 6.59–8.14) × 100,000 and 8.75 and 6.05 in males and females, respectively. Age‐specific prevalence rates did not differ among males and females in the population aged less than 49 years. The difference emerged in population aged > 50 years. This type of diagnosis was recorded for 343 patients (11 missing). 68% of these patients have a definite diagnosis, 14% likely, 11% possible, and 12% defined as suspect.

**Conclusions:**

The estimate of prevalence rates observed in this study is probably in line with the values reported in the literature for prospective prevalence studies.

## Abstract 2

The aim of this study is to conduct for the first time, an estimate of the amyotrophic lateral sclerosis prevalence in the Latium region.

## 1 INTRODUCTION

According to numerous studies, amyotrophic lateral sclerosis (ALS) incidence and prevalence widely varies between geographical areas worldwide, presuming that several factors (ancestral, genetic, environmental, and lifestyle) play an important role in the occurrence of the disease. However, methodological heterogeneity among studies determined through the years, conflicting results between European and non‐European studies.

A meta‐analysis of 44 studies covering 45 geographical areas and 11 sub‐continents, first reported in 2017, pooled estimates of ALS incidences worldwide of 1.68/100,000 PYFU (number of cases per 100,000 person‐years of follow‐up) (1.50−1.85), 1.96 (1.75–2.18) for men and 1.39 (1.21–1.56) for women, with a standardized sex ratio of 1.41 [1]. However, heterogeneity was observed between Northern Europe [1.89 (1.46−2.32)/100,000 PYFU] and East Asia [0.83 (0.42−1.24)/100,000 PYFU, China and Japan] or South Asia [0.73 (0.58−0.89)/100,000/PYFU Iran], all statistically significant (Marin et al., 2017). This study, however, reported homogeneous rates between populations from Europe, North America, and New Zealand with a pooled ALS incidence of 1.81 (1.66–1.97)/100,000 PYFU. Conversely, no data regarding prevalence rates were reported (Marin et al., 2017).

In another systematic review published in 2013 on 37 studies, a median incidence rate of 1.90 (1.37, 2.40) including Europe, North America and Asia was calculated with a difference between values from prospective and retrospective studies [2.26 (1.80, 2.60) and 1.50 (0.92, 2.00)], respectively (Chiò et al., 2013). Twenty included studies reported prevalence. According to these 20 studies, prevalence estimate was 7.89 [6.25, 7.98] in prospective studies, while retrospective studies reported a lower prevalence estimate [4.04 (3.92, 4.70)] (Chiò et al., 2013)^.^

In Europe, prevalence rates ranged between 1.1/100,000 and 11.2/100,000, with two studies from Italian regions that were among the highest in Europe (7.89/100,000 for the first and a constant increase in prevalence rates throughout the years of the study—from 5.8/100,000 on December 31, 2000, to 11.2/100,000 on December 31, 2009 for the second) (Chiò et al., 2009; Georgoulopoulou et al., 2011).

In Europe, the estimated number of prevalence ALS cases is around 40,000; in Asia, prevalence cases reported are 45,000, while for the United States, only two studies calculated the number of prevalence cases, estimating about 10,500 cases (Chiò et al., 2013).

To date, the origin of geographic difference in ALS incidence and prevalence is a matter of debate in the scientific community. Undoubtedly in European countries, where, since 1989, the birth of ALS population‐based registries (the first one in Scotland) helped researchers to develop different systematic approaches to collect data in order to obtain more accurate information (Logroscino et al., 2008; Rooney et al., 2017).

A recent study based on the National ALS Registry reported a higher ALS prevalence in the United States, with about 15,000 cases (Mehta et al., 2018).

However, all evidences converge to show that risk of ALS is different between continents and ethnicities. Geographic gradients and different ethnicities may play an active role in ALS risk (Logroscino & Piccininni, 2019).

For example, in Italy, an ecological study investigated the possible role of latitude gradient in ALS risk, underlying that the ALS incidence rates of patients born in three Southern Italian regions were significantly higher than those of persons born in northern areas (Chiò et al., 1999).

Ethnicities also seem to play a role as several studies agreed that Caucasians have a higher risk compared to non‐Caucasians (Afro‐American, Hispanic, Asian) (Logroscino & Piccininni, 2019).

In a recently pooled meta‐analysis, another important element evaluated that needs further investigations in the direction of studying differences between ethnicities is the age of onset: the variation of ALS rates with age in populations of European origin showed a peak of 6.98 and 7.77 per 100,000 person years (PY) at 71.6 and 77.4 years of age, respectively in Northern Europe and North America. In Southern and Western Europe, a peak of 7.46 and 8.17 per 100,000 PY at 74.6 and 74.9 was reported, respectively (Marin et al., 2018).

In East Asia, the peak of ALS incidence was estimated to be 74.3 years of age, which is in the same age range as that of Caucasians, but incidence rate was at 2.2 per 100,000 PY (Marin et al., 2018).

Genetic factors may have a key role in ALS risk: gene C9ORF72 is the most commonly known gene that is associated with ALS, with higher presence in Caucasians; it explains about 40% of familial case and 8% of sporadic cases. The prevalence of C9ORF72 is much lower in other ethnicities (Asian populations) (Pliner et al., 2014).

Recently, studies have attempted to estimate the projected increase of ALS cases between 2015 and 2040. ALS cases will increase from more than 222,000 to more than 376,000 globally, and this is probably due to aging populations mainly in developing countries where median age is slowly but progressively rising (Arthur et al., 2016).

ALS pathology occurs with a wide range of clinical characteristics among patients. Site of onset results in several different phenotypes and includes spinal onset, bulbar‐onset, and trunk and global body weakness onset (Raymond et al., 2019; Williams et al., 2013). The ALS patients with bulbar onset typically have a worse prognosis than spinal onset (Chiò et al., 2009).

The aim of this paper is to conduct for the first time, an estimate of the ALS prevalence in the Latium region and to characterize the different clinical forms.

## 2 MATERIALS AND METHODS

In 2014, the project “Elaboration of the Amyotrophic Lateral Sclerosis Pathology Registry in the Latium Region” was funded by the Department of Social Policies of the Latium Region, with the aims to establish an ALS pathology registry in the Latium Region and to analyze demographic and clinical characteristics, and socio‐welfare aspects of the patients.

The study was performed in Latium, a region situated in central Italy, with a land area of 17,232 km^2^. Administratively, Lazio is divided into four provinces (Frosinone, Latina, Rieti, and Viterbo) and one metropolitan (province‐level) city (Rome).

As of January 1, 2016, the population of the study area was of 5,888,472 million (2,838,616 men; 3,049,856 women).

As a first step to conduct the study, we identified clinical centers (located in hospitals and in local health authorities) in the Latium region involved in the diagnosis and treatment of ALS patients. A network of 25 centers was established, starting from the involvement of the four regional reference clinical center for ALS (located in Rome), 11 clinical hospital centers (located in all Latium provinces), and 10 local health authorities.

A study research protocol was developed and shared with the network of 25 ALS centers.

To properly estimate prevalence of ALS in Latium in 2016, the number of ALS cases—fully characterized by clinical diagnosis, date of diagnosis, life status in the year of the survey, and residence—and population data from which ALS cases were derived, were obtained (Rothman & Greenland, 1998).

Each patient was classified according to the original and revised El Escorial criteria (Brooks et al., 2000). In particular, a patient was diagnosed with ALS under the presence of upper motor neuron (UMN) and lower motor neuron (LMN) findings in four regions (bulbar, cervical, thoracic, and lumbosacral). Depending on the extent of involvement of the UMN and LMN and the number of affected regions, ALS was diagnosed as definite, probable, possible, and suspected. Site of onset refers to the body part where patient reported their first ALS symptom. The body was divided into the following four groups: (a) limb extremities included hand, arm, foot, or leg; (b) bulbar included speech and/or swallowing; c) trunk included neck, back or abdominal areas, breathing muscles; d) global body weakness.

The ALS patients diagnosed before December 31, 2016, who lived at least one day in the year 2016, and who were resident in the Latium region in 2016 were considered in this prevalence study.

The enrollment was started on July 2016 and ended on March 2017. For the present study, the research protocol was approved by the independent ethics committee of Italian National Institute of Health and an informed consent was signed by participants.

### 2.1 Statistical analysis

Population data on residents in Latium and in Italy as of January 1, 2016 were obtained from the website of the Italian National Bureau of Statistics (ISTAT, http://www.demo.istat.it). For each Latium province (Frosinone, Latina, Rome, Rieti, Viterbo), population data by gender and age were retrieved. Population data for Latium region were obtained by summing the data of Latium provinces. Crude prevalence estimates were calculated by the ratio of number of ALS cases and the respective population and by standardizing for age and sex, using the direct method and the 2016 Italian population (≥20 years) as the standard. Overall rates were adjusted for age and sex, female and male rates were adjusted for age.

Both crude prevalence estimates—for Latium region and for each Latium province—and age‐ and/or sex‐specific specific estimates were obtained. Rates were reported × 100,000 inhabitants, and 95% confidence intervals (CI) were obtained by using the formula by Tiwari et al. (2006), which is preferable in the case of rare diseases.

Moreover, for each province, standardized prevalence ratios (SPR) were calculated by the indirect method by applying European prevalence rates consisting of 28 countries—available from Arthur et al. (2016)—to gender‐ and age‐specific population data. Confidence intervals were obtained and statistical significance was judged accordingly.

Distribution of site of disease onset in gender‐ and age‐specific prevalence rates was also described.

## 3 RESULTS

The prevalence study identified 353 ALS cases (200 males and 153 females) who (i) received within December 31, 2016, a diagnosis di ALS according to El Escorial criteria; (ii) lived at least one day in the Latium region during the study period (from January 1, 2016 to December 31, 2016); and (iii) who were resident in the Latium region. The type of diagnosis was recorded for 343 patients and was missing in 11. Of these patients, 68% had a definite diagnosis, 14% likely, 11% possible, and 7% defined as suspect.

**FIGURE 1:**
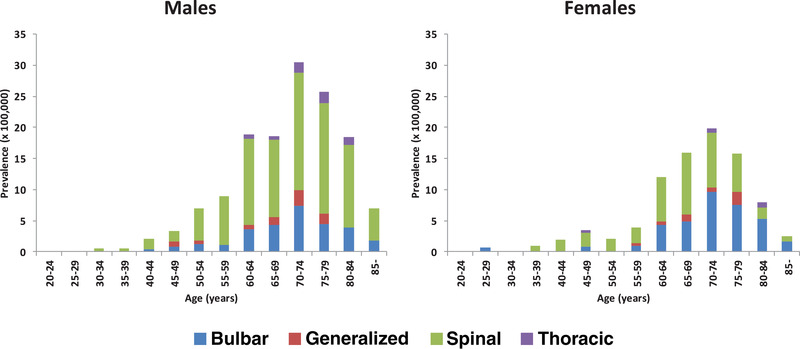
Site of onset of disease in age and gender specific ALS prevalence rates in Latium in 2016

As of January 1, 2016, there were 5,888,472 residents in Lazio. Of them, 4,815,659 (82%) were aged >= 20 years. By considering the population aged >= 20 years, the crude prevalence rate resulted as 7.33 (CI95% 6.59–8.14) × 100,000 [8.75 and 6.05 (× 100,000) in males and females, respectively]. Age‐specific prevalence rates did not differ among males and females in the population aged less than 49 years. The difference emerged in the population aged > 50 years. The increase in age‐specific prevalence rates occurred in the last 50 years for males, and the past 60 years for females. Moreover, in age classes with highest ALS prevalence rates (60–64, 65–69), male prevalence rates showed a 50% excess, respective to females rates (Table 1, Figure 1).

**TABLE 1:** Age‐ and gender‐specific crude prevalence rates in Latium in 2016

	Male			Female			Total		
Age classes (years)	ALS cases	Population	Prevalence (per 100,000)	ALS cases	Population	Prevalence (per 100,000)	ALS cases	Population	Prevalence (per 100,000)
**20–24**	0	148,216	0.00	0	136,644	0.00	0	284,860	0.00
**25–29**	0	159,678	0.00	1	153,883	0.65	1	313,561	0.32
**30–34**	1	179,047	0.56	0	177,769	0.00	1	356,816	0.28
**35–39**	2	206,549	0.97	2	210,702	0.95	4	417,251	0.96
**40–44**	5	238,788	2.09	5	250,175	2.00	10	488,963	2.05
**45–49**	9	242,265	3.71	10	256,716	3.90	19	498,981	3.81
**50–54**	16	229,645	6.97	6	245,134	2.45	22	474,779	4.63
**55–59**	18	189,934	9.48	9	207,869	4.33	27	397,803	6.79
**60–64**	32	164,568	19.44	22	183,296	12.00	54	347,864	15.52
**65–69**	32	160,921	19.89	29	182,736	15.87	61	343,657	17.75
**70–74**	37	121,504	30.45	30	146,083	20.54	67	267,587	25.04
**75–79**	30	112,859	26.58	25	146,137	17.11	55	258,996	21.24
**80–84**	14	75,652	18.51	11	112,413	9.79	25	188,065	13.29
**85 +**	4	56,891	7.03	3	119,585	2.51	7	176,476	3.97
**Total**	200	2,286,517	8.75	153	2,529,142	6.05	353	4,815,659	7.33

The age‐standardized prevalence of ALS considering population aged >= 20 years for total, females and males was 7.54 per 100,000 (95% CI 6.77–8.37), 6.18 per 100,000 (95% CI 5.24–7.24), and 9.02 per 100,000 (95% CI 7.81–10.36), respectively, using the Italian population as standard.

Population data, number of ALS cases, and crude and standardized prevalence rates by province are reported in Table 2. The Latium population was mainly resident in the province of Rome (about 73%), about 1 of 10 was resident in the province of Latina (10%) or Frosinone (9%), and few were resident in Viterbo (5%) or Rieti (3%). The majority of ALS Latium patients was resident in Rome (*n* = 290, 82.2%), some cases were resident in Latina (*n* = 23, 6.5%), Frosinone (*n* = 23, 6.5%), and Viterbo (*n* = 14, 4.0%), and very few cases were resident in Rieti (*n* = 3, 0.8%). The crude prevalence rate showed differences by province (see Table 2), with a very low rate in the province of Rieti. Standardized prevalence ratios suggested that prevalence of ALS in Lazio provinces did not differ from European estimates, except for the province of Rieti (see in Table 2), suggesting that the lower prevalence rate observed in Rieti was not fully explained by age distribution.

**TABLE 2:** Prevalence of ALS by province in Latium in 2016

Province	Population (>= 20 years)	ALS cases (*n*)	Crude prevalence rate (per 100,000)	Standardized prevalence ratio* (CI 95%)	Adjusted rate** (per 100,000) (CI 95%)
Frosinone	409491	23	5.62	0.74 (0.47–1.11)	5.50 (3.48–8.26)
Latina	466692	23	4.93	0.67 (0.43–1.01)	5.11 (3.23–7.67)
Roma	3539584	290	8.19	1.11 (0.98–1.24)	8.50 (7.55–9.54)
Rieti	132922	3	2.26	0.28 (0.06–0.83)	2.20 (0.45–6.46)
Viterbo	266970	14	5.24	0.67 (0.37–1.13)	5.14 (2.81–8.63)

* Indirect method; European population as references (Arthur et al., 2016).

** Direct method, Italian population as reference (ISTAT, http://www.demo.istat.it).

Information on site of onset was available on 342 patients (11 missing data) with the following frequencies: spinal (62.9%), bulbar (27.4%), thoracic (2.9%), generalized (5.6%), and not assessable for 4 patients (1.2%). Analysis by site of disease onset highlighted prevalence rates of ALS with bulbar onset higher in females than in males in advanced age classes (from 70 to 84 years) (Figure 1) Age‐specific prevalence rates for spinal onset were higher in males in all age classes.

## 4 DISCUSSION

The estimate of prevalence rates observed in this study is probably in line with the median value reported in the literature for seven European prospective prevalence studies (7.33 vs. 7.89 cases for 100,000 inhabitants) (Chiò et al., 2013).

In Italy, there are studies with a higher prevalence (11.2/100,000 inhabitants in the province of Modena, 10.8 per 100,000 in Sardinia, and 10.5/100,000 inhabitants in Piedmont and Val D'Aosta) (Chiò et al., 2017; Georgoulopoulou et al., 2011; Pugliatti et al., 2013) than our estimate. The reasons for this difference is that these studies have a dual source of data (cases identified by clinical centers, and cases present in current health flows such as death certificate, hospital records, and drugs prescriptions) while our study only refers to the source of clinical centers

However, our study probably underestimates the real phenomenon of ALS frequency in some provinces of Latium region. Table 2 shows a statistically significant underestimate of 72% compared with European data for the province of Rieti. The other provinces, with the exception of Rome, showed an underestimate, although not significant, and are all bordering on the other regions where there are specialist centers for ALS. It is likely that some patients will turn outside the Latium region and that they will change their place of residence especially in the early stages of illness. However, different distribution of genetic and/or environmental factors associated with onset of ALS in the provinces of Latium cannot be excluded.

As far as the clinical forms at onset, our study showed some relevant differences from those reported in the literature. In fact, the bulbar form at onset is more represented in this study (27.4%) than the frequencies reported by the other authors (22% and 17%) (Raymond et al., 2019; Williams et al., 2013). On the other hand, the spinal form at onset was less frequent in our study respective to those published (62.9% vs. 72% and 74%) (Raymond et al., 2019; Williams et al., 2013).

This difference could be attributed to three factors. In our study, the frequency of patients with an established high diagnostic level of 68% for defined ALS and 14% for likely ALS is probably high.

This may indicate that in our study, there is greater accuracy in reconstructing the clinical history of an ALS patient. However, it is difficult to make a specific comparison with other studies in the absence of data on the level of diagnostic accuracy (Chiò et al., 2009; Williams et al., 2013). Another possible difference is in the methodological profile of these studies: Ours is a prevalence study based on the creation of a network of 15 clinical centers (of which four referral ALS centers were located in Rome) involved specifically in the diagnosis and treatment of ALS patients, while in the other two studies a registry's online clinical survey module and the US Centers for Medicare & Medicaid Services Medicare longitudinal claims database were used (Chiò et al., 2009; Williams et al., 2013). At last, missing patients are likely to be long‐survivors and this would justify the lower representation of spinal patients with respect to the literature (Pupillo et al., 2014).

In our study, the ALS with bulbar onset was more frequent in females than in males in advanced age classes, while age‐specific prevalence rates for spinal onset were higher in males in all age classes (see Figure 1). This different pattern may be relevant from an etiological prospective. In a previous case‐control study performed in the province of Rome, some of the hypothesized risk factors (smoke, occupational exposure, and head injures) for ALS have been found positively associated with different patterns between bulbar and spinal ALS (Binazzi et al., 2009). However, it is reported that the incidence of ALS varied across provinces according to age, sex, and site of onset (Beghi et al., 2007).

This complex pathology is probably due to an interaction between genetic and environmental factors that must yet be identified (Al‐Chalabi & Hardiman, 2013; Wang et al., 2017). The rare frequency of ALS prevented the conduction of well‐designed case‐control studies with adequate sample size. Some authors propose a disease model in which ALS is considered to be the result of environmental risks and time acting on a pre‐existing genetic load (Al‐Chalabi & Hardiman, 2013). The future challenge for the scientific community is to combine descriptive epidemiology capable of characterizing the natural history of different phenotypes of the disease with an analytical epidemiology that avoids the contrast between the supporters of environmental theory and those of genetic theory.

## CONFLICT OF INTEREST

The authors declare no conflict of interest.

## AUTHOR CONTRIBUTIONS

Conceptualization: Nicola Vanacore and Maria Puopolo. Methodology: Maria Puopolo, Eleonora Lacorte, Flavia Mayer, and Alessandra Di Pucchio. Clinical investigation: Marialaura Santarelli, Maurizio Inghilleri, Antonio Petrucci, and Mario Sabatelli. Formal analysis: Maria Puopolo and Flavia Mayer Data curation: Ilaria Bacigalupo, Alessandra Di Pucchio, Flavia Mayer, Paola Piscopo, Alessio Crestini, Annamaria Confaloni, Fabrizio Marzolini Writing—original draft preparation: Maria Puopolo, Ilaria Bacigalupo, and Nicola Vanacore. Writing—review and editing: Maria Puopolo, Ilaria Bacigalupo, Flavia Mayer, Paola Piscopo, Eleonora Lacorte, Alessandra Di Pucchio, Annamaria Confaloni, Fabrizio Marzolini, Marialaura Santarelli, Maurizio Inghilleri, Antonio Petrucci, Mario Sabatelli, Nicola Vanacore. Supervision: Nicola Vanacore, Maria Puopolo, and Ilaria Bacigalupo. The Latium Registry Group has been involved in the clinical investigation of ALS cases. All authors have read and agreed to the published version of the manuscript.

### PEER REVIEW

The peer review history for this article is available at https://publons.com/publon/10.1002/brb3.2378.

## Data Availability

Accessibility of protocol, raw data, and programming code available upon request to corresponding author.
